# Rapid Reversal of Pulmonary Hypertension by High-Dose Thiamine Supplementation: A Case Report

**DOI:** 10.1016/j.cjco.2024.09.013

**Published:** 2024-10-04

**Authors:** Tomonari Moriizumi, Takahiro Hiraide, Mizuki Momoi, Yoshiki Shinya, Yoshikazu Kishino, Yasuyuki Shiraishi, Wataru Iai, Keisuke Matsumura, Shun Kohsaka, Masaki Ieda

**Affiliations:** a Department of Cardiology, Keio Hospital, Tokyo, Japan; b Department of Cardiology, National Hospital Organization Saitama Hospital, Wako, Japan

**Pulmonary hypertension (PH) is characterized by progressive right heart failure and a poor prognosis. A 50-year-old man with schizophrenia and poor nutrition status presented with palpitations, dyspnea at rest, and shock. Right heart catheterization demonstrated an elevated mean pulmonary arterial pressure (PAP) of 40 mm Hg and normal pulmonary artery wedge pressure, indicating precapillary PH. Despite the initiation of catecholamines and diuretics, the patient’s condition remained refractory, and shock persisted. Subsequent thiamine supplementation resulted in a rapid reduction of PAP, to 17 mm Hg. Prompt recognition of thiamine deficiency in precapillary PH is essential to prevent a fatal outcome.**

## Case Presentation

**Figure 1:**
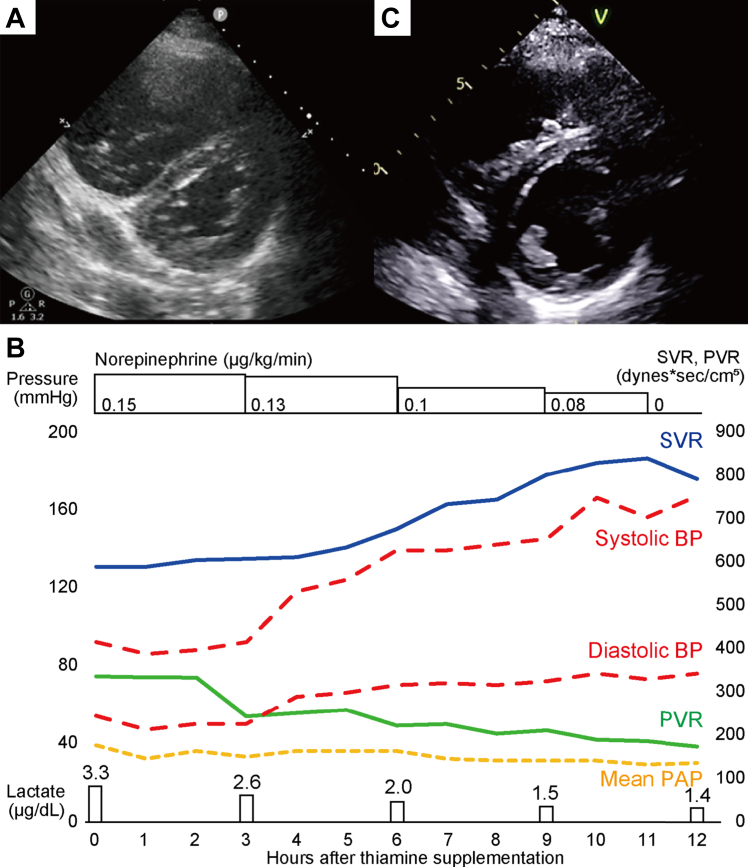
Echocardiography and clinical course. (**A**) Echocardiography (parasternal view, short axis) at admission. (**B**) Overview of clinical course after the thiamine supplementation. The **blue line** indicates systemic vascular resistance (SVR); the **green line** indicates pulmonary vascular resistance (PVR); the **dashed red lines** indicate systolic and diastolic systemic blood pressure (BP); and the **dashed yellow line** indicates mean pulmonary artery pressure (PAP). (**C**) Echocardiography (parasternal view, short axis) at discharge.

A 50-year-old man was admitted to the hospital for palpitations and progressive dyspnea over 3 weeks (World Health Organization functional class IV). He had a longstanding history of schizophrenia and diabetes mellitus. His medications included the following: bromazepam 12 mg/d; anafranil 60 mg/d; gliclazide 20 mg/d; metformin 1000 mg/d; ipragliflozin 50 mg/d; and pitavastatin 1 mg/d. He had an unbalanced diet, consuming only polished rice for 1 meal each day, and he had no history of drinking alcohol. He had no family history of cardiac disease, including pulmonary arterial hypertension. His blood pressure at arrival was 77/64 mm Hg, and his pulse rate was 120 beats/min. He had marked bilateral extremity edema. Laboratory tests demonstrated significant liver damage, kidney injury, and elevation of the levels of B-type natriuretic peptide (1023.8 pg/mL; normal range, ≤ 14.6 pg/mL), troponin-T (0.105 ng/mL; normal range, < 0.014 ng/mL); and lactate (7 mmol/L; normal range, 0.5-2.2 mmol/L). All serologic tests related to PH, including thyroid-function, autoimmune antibody panels, HIV serology, and thrombophilia screening, were negative. Echocardiography at admission demonstrated the following: a preserved left-ventricular ejection fraction of 57.5%; right-ventricular enlargement; a flattened interventricular septum; a peak tricuspid regurgitant pressure gradient of 39 mm Hg; estimated right-ventricular systolic pressure of 54 mm Hg; a pulmonary artery acceleration time of 90 msec; a tricuspid annular systolic velocity of 9.9 m/s; and a tricuspid annular plane systolic excursion of 10.8 mm (Fig. 1A). An electrocardiogram demonstrated right-axial deviation, and no pulmonary P wave. T-wave inversion was present in precordial leads V1-V3. Contrast-enhanced computed tomography ruled out pulmonary embolism and congenital heart disease. Right heart catheterization with norepinephrine (0.15 μg/kg/min) demonstrated the following: a right-atrial pressure of 18 mm Hg; right-ventricular pressure of 49/20 mm Hg; mean PAP of 40 mm Hg; a pulmonary artery wedge pressure (PAWP) of 10 mm Hg; cardiac output of 3.92 L/min; a cardiac index of 2.05 L/min/m^2^; pulmonary vascular resistance (PVR) of 612 dynes/s/cm^5^; and a systemic vascular resistance (SVR) of 1000 (normal range, 1200 ± 200) dynes s/cm^5^. No significant arteriovenous shunts were detected by oximetry. These data suggested that the patient had high-risk precapillary PH.

Although dobutamine (3 μg/kg/min), norepinephrine (0.15 μg/kg/min), and continuous hemodialysis were administered, the patient’s acute kidney injury and congestive right heart failure persisted. Consequently, he was transferred to the intensive-care unit on day 5. His systemic blood pressure was low, at 88/50 (mean: 61) mm Hg, and his SVR was decreased to approximately 600 dynes s/cm^5^. He had no signs of infection. Given his unbalanced diet, low SVR, and elevated serum lactate level, we suspected thiamine deficiency. The serum thiamine levels were decreased to 2.4 μg/dL (normal range, 2.8-5.8 μg/dL by the liquid chromatography–mass spectrometry method), prompting the initiation of intravenous thiamine supplementation at 200 mg/d on day 6 after admission. After he received the high-dose thiamine treatment, his blood pressure improved substantially within a few hours, and norepinephrine was discontinued after 12 hours (Fig. 1B). On the day 7, continuous hemodialysis was discontinued, and his mean PAP improved to 21 mm Hg. Thiamine supplementation was completed on day 11. On day 24, right heart catheterization demonstrated normal hemodynamics, with a right-atrial pressure of 4 mm Hg, a right-ventricular pressure of 28/7 mm Hg, a mean PAP of 17 mm Hg, a PAWP of 8 mm Hg, a cardiac output of 5.42 L/min, a cardiac index of 2.85 L/min/m^2^, a PVR of 133 dynes s/cm^5^, and an SVR of 1122 dynes s/cm^5^ without pulmonary vasodilators. Echocardiography demonstrated a preserved left-ventricular ejection fraction of 58.1%, resolution of the flattened interventricular septum, a peak tricuspid regurgitant pressure gradient of 16 mm Hg, an estimated right-ventricular systolic pressure of 19 mm Hg, a pulmonary artery acceleration time of 103 msec, s tricuspid annular systolic velocity of 10.2 m/s, and a tricuspid annular plane systolic excursion of 20.8 mm (Fig. 1C). On day 27, he was discharged, after receiving nutritional counselling. His symptoms have been stable for 6 months. His hemodynamic changes are detailed in Table 1.

**Table 1:** Changes of hemodynamics

Time	Mean RAP, mm Hg	PAP, mm Hg (mean)	Mean PAWP, mm Hg	CO, L/min		CI, L/min/m^2^		PVR, dynes s/cm^5^		SVR, dynes s/cm^5^		SaO2, %	SvO2, %
				Thermo	Fick	Thermo	Fick	Thermo	Fick	Thermo	Fick		
On admission∗	18	57/28(40)	10	3.92	3.49	2.05	1.83	612	687	1000	1123	92.7	56.3
Day 5†	12	54/31(40)	13	—	6.50	—	3.40	—	332	—	603	95.8	74.2
Day 24	4	29/8(17)	8	5.42	5.42	2.85	2.85	133	133	1122	1122	97.5	73.2

Pulmonary artery pressure (PAP) was described as systolic / diastolic (mean) pressure.

CI, cardiac index; CO, cardiac output; Fick, Fick method; PAWP, pulmonary artery wedge pressure; PVR, pulmonary vascular resistance; RAP, right-atrial pressure; SaO2, arterial oxygen saturation; SvO2, mixed venous oxygen saturation; SVR, systemic vascular resistance; Thermo, thermodilution method.

∗ Right heart catheterization was performed with norepinephrine 0.15 μg/kg/min, and 6 L/min oxygen.

† Right heart catheterization was performed with norepinephrine 0.15 μg/kg/min, dobutamine 3 μg/kg/min, and 6 L/min oxygen.

## Discussion

Thiamine is a water-soluble vitamin and an essential coenzyme in the conversion of pyruvate to acetyl coenzyme A (CoA) in glycolysis. Acetyl-CoA enters the mitochondria to produce adenosine triphosphate (ATP). Depletion of intracellular ATP leads to peripheral vasodilation, reduced SVR, and arteriovenous shunting, eventually causing high-output heart failure, known as wet beriberi.^1^ In previous reports, most thiamine deficiency–related pulmonary hypertension was caused by a high level of cardiac-output failure of the left ventricle, with intact PVR.^2^ However, the thiamine-deficiency disruption of the conversion of pyruvate to acetyl CoA leads to lactic acidosis. Moreover, ATP depletion and reactive oxygen species production occur when the tricarboxylic acid cycle fails, resulting in decreased nitric oxide activity, pulmonary vasoconstriction, and increased PVR, leading to precapillary PH.^3^ A case of PH due to thiamine deficiency has been reported from Japan.^4^ Our patient exhibited an elevated mean PAP, an increased PVR, a normal PAWP, and a low cardiac-output level, consistent with precapillary PH without high-output status. These findings suggest that thiamine deficiency can present as severe, transient precapillary PH with right-sided shock. Further research is warranted to elucidate the molecular mechanism underlying the development of wet beriberi, and precapillary PH, in the context of thiamine deficiency.

The tissue storage of thiamine is limited, and it has a half-life of 10–20 days, making deficiency possible within a few weeks. Accelerated excretion by use of diuretics, and diabetes mellitus, also may contribute to this deficiency.^4^ Given that only 0.8% of the body’s total thiamine is present in the blood, serum thiamine levels may not accurately reflect whole-body thiamine status.^1^ Currently, no definitive biomarker or normal range for diagnosing thiamine deficiency has been established.^3^ In this patient, serum thiamine levels were decreased only mildly, suggesting that his overall body stores were insufficient. Previous reports have documented severe PH despite mildly reduced serum thiamine levels, consistent with our findings.^5^ Therefore, a therapeutic diagnosis approach may be necessary to detect thiamine deficiency, as blood tests alone may not be sufficient.

In terms of the treatment, thiamine should be administered as early as possible, with the dose ranging from 100 mg intravenously once daily to 400 mg intravenously twice daily.^6^ In this patient, although the patient required high-dose catecholamine within a few days, and the condition became so severe that extracorporeal circulation was considered, dramatic improvement was achieved by early initiation of thiamine supplementation. Given the minimal risks associated with thiamine administration, prompt treatment should be considered in patients with PH, even if serum thiamine levels are decreased only slightly.

### Limitations

The limitations of this case include the potential presence of additional deficiency factors that may have contributed to the development of PH. For instance, reports have been made of reversible severe PH associated with vitamin-C deficiency.^7^ Although the condition in our case improved with thiamine supplementation, the possibility that vitamin-C deficiency also could have played a role in the pathogenesis of PH is plausible.

### Conclusions

We had a case of severe hemodynamic instability in a patient with PH, which normalized following high-dose thiamine supplementation. Thiamine deficiency should be considered as a potential underlying cause of severe PH with low SVR and elevated serum lactate concentrations, and thiamine supplementation should be initiated promptly.

**Novel Teaching Points**

• Thiamine deficiency can lead to severe, reversible PH.
• Thiamine deficiency should be considered in patients with PH, especially in those with a compromised nutritional status, such as individuals with schizophrenia.
• When thiamine deficiency is suspected, prompt initiation of thiamine supplementation is recommended, even if serum thiamine levels are reduced only mildly.
